# Complete genome sequence of *Halorhabdus utahensis* type strain (AX-2^T^)

**DOI:** 10.4056/sigs.31864

**Published:** 2009-11-22

**Authors:** Iain Anderson, Brian J. Tindall, Helga Pomrenke, Markus Göker, Alla Lapidus, Matt Nolan, Alex Copeland, Tijana Glavina Del Rio, Feng Chen, Hope Tice, Jan-Fang Cheng, Susan Lucas, Olga Chertkov, David Bruce, Thomas Brettin, John C. Detter, Cliff Han, Lynne Goodwin, Miriam Land, Loren Hauser, Yun-Juan Chang, Cynthia D. Jeffries, Sam Pitluck, Amrita Pati, Konstantinos Mavromatis, Natalia Ivanova, Galina Ovchinnikova, Amy Chen, Krishna Palaniappan, Patrick Chain, Manfred Rohde, Jim Bristow, Jonathan A. Eisen, Victor Markowitz, Philip Hugenholtz, Nikos C. Kyrpides, Hans-Peter Klenk

**Affiliations:** 1 DOE Joint Genome Institute, Walnut Creek, California, USA; 2 DSMZ - German Collection of Microorganisms and Cell Cultures GmbH, Braunschweig, Germany; 3 Los Alamos National Laboratory, Bioscience Division, Los Alamos, New Mexico, USA; 4 Oak Ridge National Laboratory, Oak Ridge, Tennessee, USA; 5 Biological Data Management and Technology Center, Lawrence Berkeley National Laboratory, Berkeley, California, USA; 6 Lawrence Livermore National Laboratory, Livermore, California, USA; 7 HZI - Helmholtz Centre for Infection Research, Braunschweig, Germany; 8 University of California Davis Genome Center, Davis, California, USA

**Keywords:** halophile, free-living, non-pathogenic, aerobic, euryarchaeon, *Halobacteriaceae*

## Abstract

*Halorhabdus utahensis* Wainø *et al*. 2000 is the type species of the genus, which is of phylogenetic interest because of its location on one of the deepest branches within the very extensive euryarchaeal family *Halobacteriaceae*. *H. utahensis* is a free-living, motile, rod shaped to pleomorphic, Gram-negative archaeon, which was originally isolated from a sediment sample collected from the southern arm of Great Salt Lake, Utah, USA. When grown on appropriate media, *H. utahensis* can form polyhydroxybutyrate (PHB). Here we describe the features of this organism, together with the complete genome sequence, and annotation. This is the first complete genome sequence of the a member of halobacterial genus *Halorhabdus*, and the 3,116,795 bp long single replicon genome with its 3027 protein-coding and 48 RNA genes is part of the *** G****enomic* *** E****ncyclopedia of* *** B****acteria and* *** A****rchaea * project.

## Introduction

Strain AX-2^T^ (= DSM 12940 = JCM 11049) is the type strain of the species *Halorhabdus utahensis,* and represents one of only two species currently assigned to the genus *Halorhabdus* [1]. Strain AX-2^T^ was first described by Wainø *et al*. in 2000 [1] as Gram-negative, motile and extremely pleomorphic organism. The organism is of interest because of its position in the tree of life, where the genera *Halorhabdus* and *Halomicrobium* constitute one of the deepest branches within the large euryarchaeal family *Halobacteriaceae*. Here we present a summary classification and a set of features for *H. utahensis* strain AX-2^T^ together with the description of the complete genomic sequencing and annotation.

### Classification and features

Only one other 16S rRNA encoding sequence has been deposited in the INSDC databases with a similarity of greater than 97% to that of strain AX-2^T^. That sequence belongs to the other species classified in the genus *Halorhabdus, H. tiamatea*, which was isolated from a sample of the brine-sediment interface of the Shaban Deep in the northern Red Sea [2]. With 95% sequence identity, strain T4.2 (AJ270232), a halophilic archaeon that is neither validly published nor preserved in any collection [3] is the next cultivated neighbor of *H. utahensis* strain AX-2^T^. Screening of environmental genomic samples and surveys reported at the NCBI BLAST server indicated no closely related phylotypes (>91% sequence similarity) can be linked to the species or genus.

**Figure 1:**
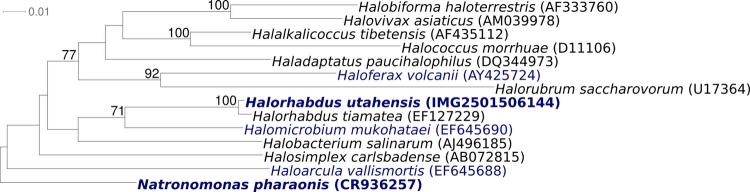
Phylogenetic tree highlighting the position of *H. utahensis* strain AX-2^T^ with a selection of type strains of the family *Halobacteriaceae*, inferred from 1,433 aligned 16S rRNA characters [4,5] under the maximum likelihood criterion [6]. The tree was rooted with *Natronomonas pharaoensis*, the deepest branching member of the family *Halobacteriaceae*. The branches are scaled in terms of the expected number of substitutions per site. Numbers above branches are support values from 1,000 bootstrap replicates, if larger than 60%. Lineages with type strain genome sequencing projects registered in GOLD [7] are shown in blue, published genomes in bold, e.g. the GEBA organism *Halomicrobium mukohataei* [8].

Figure 1 shows the phylogenetic neighborhood of *H. utahensis* strain AX-2^T^ in a 16S rRNA based tree. The sequence of the unique 16S rRNA gene is identical with the previously published 16S rRNA sequence generated from DSM 12940 (AF071880).

**Figure 2:**
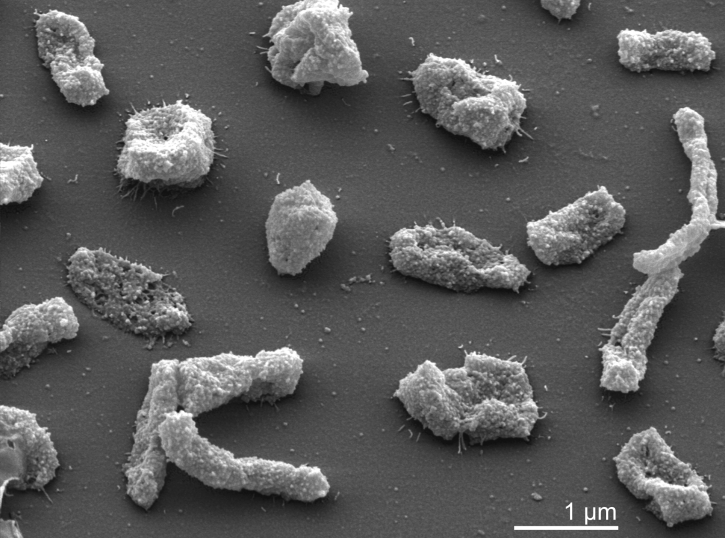
Scanning electron micrograph of *H. utahensis* strain AX-2^T^

*H. utahensis* strain AX-2^T^ is rod shaped, but may also form pleomorphic cells (Table 1 and Figure 2). Cells are motile by a single flagellum. Strain AX-2^T^ does not require amino acids for growth and will grow on defined medium containing a nitrogen source, using a single carbon source. Cells may grow anaerobically on glucose by fermentation. Polyhydoxybutyrate inclusions are formed on appropriate media. Spores or other resting stages are not produced. Oxidase and catalase are positive. Cells lyse in distilled water. Gelatin and starch were not hydrolyzed. Proteases not produced and urea was not hydrolyzed; aesculin is hydrolyzed. Esterase, lipase and glucosidase are produced. Arginine dihydrolase is not produced, and consequently arginine does not support anaerobic growth. Ornithine and lysine are not decarboxylated. Growth on glucose, xylose and fructose. Nitrate is reduced to nitrite, but does not support growth [1].

**Table 1:** Classification and general features of *H. utahensis* strain AX-2^T^ in accordance with the MIGS recommendations [9]

**MIGS ID**	**Property**	**Term**	**Evidence code**
	Classification	Domain *Archaea*	TAS [10]
		Phylum *Euryarchaeota*	TAS [11,12]
		Class *Halobacteria*	TAS [13]
		Order *Halobacteriales*	TAS [14]
		Family *Halobacteriaceae*	TAS [15
		Genus *Halorhabdus*	TAS [1]
		Species *Halorhabdus utahensis*	TAS [1]
		Type strain AX-2	TAS [1]
	Gram stain	negative	TAS [1]
	Cell shape	rod to pleomorphic	TAS [1]
	Motility	motile by a single flagellum	TAS [1]
	Sporulation	nonsporulaing	TAS [1]
	Temperature range	mesophile, 15-55°C	TAS [1]
	Optimum temperature	50°C	TAS [1]
	Salinity	halophile, at least 9% (w/v) NaCl, maximum 30%, with an optimum at 27%	TAS [1]
MIGS-22	Oxygen requirement	primarily aerobe; facultatively anaerobic growth *via* glucose fermentation	TAS [1]
	Carbon source	glucose, xylose and fructose	TAS [1]
	Energy source	carbohydrates	NAS
MIGS-6	Habitat	aquatic	TAS [1]
MIGS-15	Biotic relationship	free living	NAS
MIGS-14	Pathogenicity	none	NAS
	Biosafety level	1	TAS [16]
	Isolation	sediment of Great Salt Lake, Utah	TAS [1]
MIGS-4	Geographic location	sediment of Great Salt Lake, Utah	TAS [1]
MIGS-5	Sample collection time	before 2000	TAS [1]
MIGS-4.1 MIGS-4.2	Latitude, Longitude	41.177, -112.502	NAS
MIGS-4.3	Depth	sea level	TAS [1]
MIGS-4.4	Altitude	not reported	

Evidence codes - IDA: Inferred from Direct Assay (first time in publication); TAS: Traceable Author Statement (i.e., a direct report exists in the literature); NAS: Non-traceable Author Statement (i.e., not directly observed for the living, isolated sample, but based on a generally accepted property for the species, or anecdotal evidence). These evidence codes are from the Gene Ontology project [17]. If the evidence code is IDA, then the property was directly observed for a living isolate by one of the authors or an expert mentioned in the acknowledgements.

### Chemotaxonomy

Menaquinones are the sole respiratory lipoquinones of *H. utahensis* strain AX-2^T^. Both MK-8 and MK-8 (VIII-H_2_) are present. The lipids are based on diphytanyl ether lipids. The major phospholipids are the C_20_ diphytanyl ether analogues of phosphatidylglycerol and methyl-phosphatidylglycerophosphate (typical of all members of the family *Halobacteriaceae*), the diether analogue of phosphatidylglycerol sulphate is absent [1]. Two glycolipids have been reported with R_f_ values consistent with their identification as a triglycosyl diphytanyl ether and the sulfated derivative, sulfated triglycosyl diphytanyl. The structures of these two lipids have not been elucidated [1]. The pigments responsible for the red color of the cells have not been recorded, but it may be predicted that they are carotenoids, probably bacterioruberins. Outer cell layers are probably proteinaceous. The presence of peptidoglycan has not been investigated, but is generally absent from members of this family *Halobacteriaceae.*

## Genome sequencing and annotation

### Genome project history

This organism was selected for sequencing on the basis of each phylogenetic position, and is part of the *** G****enomic* *** E****ncyclopedia of* *** B****acteria and* *** A****rchaea * project. The genome project is deposited in the Genome OnLine Database [7] and the complete genome sequence in GenBank. Sequencing, finishing and annotation were performed by the DOE Joint Genome Institute (JGI). A summary of the project information is shown in Table 2.

**Table 2:** Genome sequencing project information

MIGS ID	Property	Term
MIGS-31	Finishing quality	Finished
MIGS-28	Libraries used	Three genomic libraries: two Sanger libraries (8 kb pMCL200 and fosmid pcc1Fos) and one 454 pyrosequence standard library
MIGS-29	Sequencing platforms	ABI3730, 454 GS FLX
MIGS-31.2	Sequencing coverage	8.3x Sanger; 21.2× pyrosequence
MIGS-30	Assemblers	Newbler version 1.1.02.15, phrap
MIGS-32	Gene calling method	Prodigal, GenePRIMP
	INSDC ID	CP001687
	Genbank Date of Release	August 27, 2009
	GOLD ID	Gc01053
	NCBI project ID	29305
	Database: IMG-GEBA	2501416929
MIGS-13	Source material identifier	DSM 12940
	Project relevance	Tree of Life, GEBA

### Growth conditions and DNA isolation

*H. utahensis* strain AX-2^T^, DSM 12940, was grown in DSMZ medium 927 (*H. utahensis* medium) [18] at 40°C. DNA was isolated from 1-1.5 g of cell paste using a Qiagen Genomic 500 DNA Kit (Qiagen, Hilden, Germany) as described in Wu *et al.* [19].

### Genome sequencing and assembly

The genome was sequenced using a combination of Sanger and 454 sequencing platforms. All general aspects of library construction and sequencing performed at the JGI can be found on the JGI website (http://www.jgi.doe.gov/). 454 Pyrosequencing reads were assembled using the Newbler assembler, version 1.1.02.15 (Roche). Large Newbler contigs were broken into 3,474 overlapping fragments of 1,000 bp and entered into assembly as pseudo-reads. The sequences were assigned quality scores based on Newbler consensus q-scores with modifications to account for overlap redundancy and to adjust inflated q-scores. A hybrid 454/Sanger assembly was made using the parallel phrap assembler (High Performance Software, LLC). Possible mis-assemblies were corrected with Dupfinisher or transposon bombing of bridging clones [20]. Gaps between contigs were closed by editing in Consed, custom primer walk or PCR amplification. A total of 212 Sanger finishing reads were produced to close gaps, to resolve repetitive regions, and to raise the quality of the finished sequence. The final assembly consists of 26,545 Sanger and 382,722 pyrosequence (454) reads. Together all sequence types provided 29.5× coverage of the genome. The error rate of the completed genome sequence is less than 1 in 100,000.

### Genome annotation

Genes were identified using Prodigal [21] as part of the Oak Ridge National Laboratory genome annotation pipeline, followed by a round of manual curation using the JGI GenePRIMP pipeline (http://geneprimp.jgi-psf.org/) [22]. The predicted CDSs were translated and used to search the National Center for Biotechnology Information (NCBI) nonredundant database, UniProt, TIGRFam, Pfam, PRIAM, KEGG, COG, and InterPro databases. Additional gene prediction analysis and functional annotation was performed within the Integrated Microbial Genomes Expert Review platform (http://img.jgi.doe.gov/er) [23].

## Genome properties

**Figure 3:**
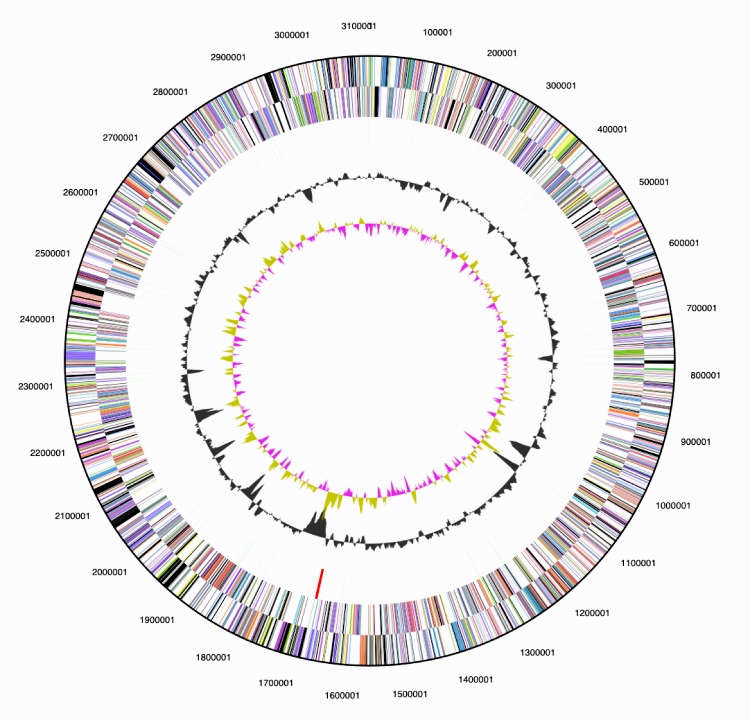
**Graphical circular map of the genome.** From outside to the center: Genes on forward strand (color by COG categories), Genes on reverse strand (color by COG categories), RNA genes (tRNAs green, rRNAs red, other RNAs black), GC content, GC skew.

The genome is 3,116,795 bp long and comprises one main circular chromosome with a 62.9% GC content (Table 3 and Figure 3). Of the 3,075 genes predicted, 3,027 were protein coding genes, and 48 RNAs; 29 pseudogenes were also identified. The majority of the protein-coding genes (60.5%) were assigned with a putative function, while those remaining were annotated as hypothetical proteins. The distribution of genes into COGs functional categories is presented in Table 4.

**Table 3:** Genome statistics

**Attribute**	**Value**	**% of Total**
Genome size (bp)	3,116,795	100.00%
DNA Coding region (bp)	2,768,833	88.83%
DNA G+C content (bp)	1,960,463	62.90%
Number of replicons	1	
Extrachromosomal elements	0	
Total genes	3,075	100.00%
RNA genes	48	1.59%
rRNA operons	1	
Protein-coding genes	3,027	98.30%
Pseudo genes	29	1.90%
Genes with function prediction	1,860	60.47%
Genes in paralog clusters	473	15.38%
Genes assigned to COGs	1,946	63.28%
Genes assigned Pfam domains	1,918	62.37%
Genes with signal peptides	705	22.93%
Genes with transmembrane helices	782	25.43%
CRISPR repeats	2	

**Table 4:** Number of genes associated with the general COG functional categories

**Code**	**Value**	**% age**	**Description**
J	157	5.2	Translation, ribosomal structure and biogenesis
A	1	0.0	RNA processing and modification
K	120	3.9	Transcription
L	115	3.8	Replication, recombination and repair
B	3	0.0	Chromatin structure and dynamics
D	26	0.8	Cell cycle control, mitosis and meiosis
Y	0	0.0	Nuclear structure
V	41	1.3	Defense mechanisms
T	121	4.0	Signal transduction mechanisms
M	82	2.7	Cell wall/membrane biogenesis
N	33	1.0	Cell motility
Z	0	0.0	Cytoskeleton
W	0	0.0	Extracellular structures
U	25	0.8	Intracellular trafficking and secretion
O	95	3.1	Posttranslational modification, protein turnover, chaperones
C	147	4.8	Energy production and conversion
G	107	3.5	Carbohydrate transport and metabolism
E	165	5.4	Amino acid transport and metabolism
F	65	2.1	Nucleotide transport and metabolism
H	106	3.5	Coenzyme transport and metabolism
I	42	1.4	Lipid transport and metabolism
P	122	4.0	Inorganic ion transport and metabolism
Q	24	0.8	Secondary metabolites biosynthesis, transport and catabolism
R	0	10.9	General function prediction only
S	214	7.1	Function unknown
-	1,081	35.7	Not in COGs
